# HIV Pre-exposure Prophylaxis Medication Sharing Among HIV-Negative Men Who Have Sex With Men

**DOI:** 10.1001/jamanetworkopen.2020.16256

**Published:** 2020-09-11

**Authors:** Gordon Mansergh, Kenneth Mayer, Sabina Hirshfield, Rob Stephenson, Patrick Sullivan

**Affiliations:** 1 Division of HIV/AIDS Prevention, Centers for Disease Control and Prevention, Atlanta, Georgia; 2 The Fenway Institute, Fenway Health, Boston, Massachusetts; 3 Department of Medicine, SUNY Downstate Health Sciences University, Brooklyn, New York; 4 Department of Systems, Population, and Leadership, School of Nursing, University of Michigan, Ann Arbor; 5 Department of Epidemiology, Rollins School of Public Health, Emory University, Atlanta, Georgia

## Abstract

This cross-sectional study used data from the M-Cubed randomized clinical trial to assess the prevalence of pre-exposure prophylaxis (PrEP) medication sharing among HIV-negative men who have sex with men.

## Introduction

When used as recommended by clinicians, pre-exposure prophylaxis (PrEP) is an effective and clinically recommended daily medication for prevention of HIV infection among sexually active men and women who do not consistently use condoms during sexual intercourse.^1^ Clinicians and their patients can decide whether PrEP is warranted and appropriate to prevent HIV infection.

Despite clinical guidelines on PrEP, some patients share their medication. Before the US Food and Drug Administration approval of antiretroviral therapy (ART) for HIV PrEP^2^ and the release of the US Centers for Disease Control and Prevention’s updated clinical guidelines on PrEP for prevention of HIV infection,^1^ research found that small proportions of HIV-negative men who have sex with men (MSM) who used ART in off-label strategies of preexposure (2% of MSM) and postexposure (4% of MSM) prophylaxis shared medication.^3^ A lower educational level, that is, having a high school diploma or less (adjusted odds ratio [aOR], 2.7 [95% CI, 1.0-7.2]; *P* = .05) vs some post–high school education and a college degree or more (aOR, 9.3; 95% CI, 1.1-76.9) was associated with sharing postexposure prophylaxis medication but not with sharing PrEP medication.^4^

These findings underscore the importance of ongoing surveillance to better understand the prevalence of sharing nonprescribed, daily oral PrEP medication and to develop strategies to address this public health issue. Given the rapidly increasing use of PrEP medication by MSM,^5^ it is critical for clinicians to monitor PrEP use to ensure its safety and effectiveness, appropriate use, and to minimize complications and the potential development of PrEP-resistant HIV strains.

## Methods

The protocol for this cross-sectional study was approved by the institutional review board of Emory University. Participants provided written informed consent to enroll in the study. This report adheres to the Strengthening the Reporting of Observational Studies in Epidemiology (STROBE) reporting guideline.

We analyzed baseline data from the 2018 M-Cubed Study, a randomized clinical trial of the use of a mobile app for prevention of HIV infection among sexually active MSM in Atlanta, Georgia; Detroit, Michigan; and New York City, New York.^6^ Participants were asked the following question: “Are you currently taking PrEP to prevent HIV?” If the answer was yes, a follow-up question was asked: “Have you ever shared your PrEP medication with others?” (yes/no). An unpaired, 2-sided χ^2^ test was used for the bivariate analysis, with *P < *.05 considered statistically significant. Multivariable logistic regression analyses were controlled for demographic characteristics (race/ethnicity, age group, educational level, and city) to assess for differences in individuals who use and share PrEP medication, with results reported as adjusted odds ratios (aORs) with 95% CIs.

## Results

This cross-sectional study included 755 HIV-negative MSM (mean [SD] age of 33.7 [11.2] years; 399 White individuals [52.9%], 146 Black/African American individuals [19.3%], 95 Hispanic/Latino individuals [12.6%], and 115 [15.2%] self-identified as other or mixed race/ethnicity). At baseline assessment, 243 individuals (32.2%) reported that they were currently receiving PrEP medication (Table). Of these individuals, 26 (10.7%) reported sharing PrEP medication, and only 12 (5.0%) had ever used PrEP on demand (ie, use of PrEP before and after sex). Multivariable logistic regression analyses adjusted for race/ethnicity, age group, educational level, and city found that MSM aged 30 to 39 years (vs those aged ≥40 years) had nearly twice the odds of reporting current PrEP use (aOR, 1.70 [95% CI, 1.11-2.61]; *P* = .02). Men with a higher educational attainment (vs men with ≤post–high school education) were more likely to report current PrEP use (4-year college degree: aOR, 1.54 [95% CI, 1.04-2.29]; *P* = .03 and >4-year college degree: aOR, 2.04 [95% CI, 1.34-3.10]; *P* = .001). Men from Detroit, Michigan (vs Atlanta, Georgia) were less likely to report current use of PrEP medication (aOR, 0.64 [95% CI, 0.43-0.97]; *P* = .03), with no differences in current use of the medication found between participants from New York City, New York, and Atlanta, Georgia. Among current PrEP users, sharing PrEP medication was more frequently reported by younger men (aged 18-29 years: aOR, 12.19 [95% CI, 1.43-103.86]; *P* = .02; aged 30-39 years: 9.78 [95% CI, 1.19-80.23]; *P* = .03) vs older men (aged ≥40 years) and by those with some graduate education (aOR, 3.67 [95% CI, 1.06-12.73]; *P* = .04) compared with some post–high school education or less.

**Table.** Characteristics of HIV-Negative MSM Using and Sharing PrEP Medication in a Diverse Sample From 3 US Cities, 2018

Characteristic	Total No. (%)	PrEP users				PrEP users who share medication			
		No. (%)	χ^2^ *P* value^a^	aOR (95% CI)	aOR *P* value	No. (%)	χ^2^ *P* value^a^	aOR (95% CI)	aOR *P* value
Overall	755 (100)	243 (32.2)		NA	NA	26 (10.7)		NA	NA
Race/ethnicity									
Latino/Hispanic	95 (12.6)	28 (29.5)	.13	1.33 (0.85-2.10)	.21	5 (17.9)	.29	0.27 (0.07-1.09)	.07
Black/African American	146 (19.3)	38 (26.0)		0.80 (0.51-1.25)	.33	2 (5.4)		0.34 (0.07-1.66)	.18
Other/mixed	115 (15.2)	45 (33.1)		0.74 (0.45-1.23)	.25	3 (6.7)		1.23 (0.37-4.11)	.73
White	399 (52.9)	132 (39.1)		1 [Reference]	NA	16 (12.1)		1 [Reference]	NA
Age group, y									
18-29	341 (45.2)	103 (30.2)	.04	1.28 (0.84-1.93)	.25	12 (11.8)	.03	12.19 (1.43-103.86)	.02
30-39	231 (30.6)	89 (38.5)		1.70 (1.11-2.61)	.02	13 (14.6)		9.78 (1.19-80.23)	.03
≥40	183 (24.2)	51 (27.9)		1 [Reference]	NA	1 (2.0)		1 [Reference]	NA
Educational level									
≤Some post–high school	262 (34.8)	62 (23.7)	<.001	1 [Reference]	NA	4 (6.6)	.01	1 [Reference]	NA
4-Year college degree	280 (37.2)	96 (34.3)		1.54 (1.04-2.29)	.03	4 (6.3)		0.73 (0.19-2.86)	.65
>4-Year college degree	211 (28.0)	85 (40.3)		2.04 (1.34-3.10)	.001	16 (18.8)		3.67 (1.06-12.73)	.04
City/MSA									
Atlanta, Georgia	269 (35.6)	88 (32.7)	<.01	1 [Reference]	NA	8 (9.1)	.78	1 [Reference]	NA
Detroit, Michigan	226 (39.9)	56 (24.8)		0.64 (0.43-0.97)	.03	6 (10.7)		0.75 (0.23-2.52)	.65
New York City, New York	260 (34.4)	99 (38.1)		1.15 (0.79-1.68)	.46	12 (12.2)		1.39 (0.50-3.87)	.53

Abbreviations: aOR, adjusted odds ratio; MSA, metropolitan statistical area; MSM, men who have sex with men; NA, not applicable; PrEP, pre-exposure prophylaxis.

^a^ *P* < .05 indicated statistical significance for the χ^2^ test.

## Discussion

Increasing numbers of MSM use daily oral PrEP medication. In this study, more than 1 in 10 current PrEP users shared their PrEP medication. Regular assessment of the extent and the context of PrEP medication sharing could aid clinicians in creating messages to discourage medication sharing while promoting prescribed and clinically monitored use of PrEP. This study is limited by its use of self-reported information from MSM living in 3 US cities. Additional research is needed on factors that facilitate PrEP medication sharing, such as cost-savings, convenience, unawareness of potential consequences, and other factors. Younger and highly educated men, in particular, could benefit from messaging about the potential consequences of sharing PrEP medication.
